# Decreased serum TIMP4 levels in patients with rheumatoid arthritis

**DOI:** 10.1515/biol-2022-1037

**Published:** 2025-04-25

**Authors:** Jinyu Chen, Yanyan Fan, Shengyu Cui, Haiping Zhang, Ziliang Yu, Yali Jiang, Xiaogang Zhou

**Affiliations:** Department of Orthopedics, The Second Affiliated Hospital of Nantong University, No. 666, Shengli Road, Nantong, 226001, China; Department of Orthopedics, Ili & Jiangsu Joint Institute of Health, Ili 835100, China; Department of Orthopedics, Nantong Hospital to Nanjing University of Chinese Medicine, Nantong, 226001, China; Depatement of Orthopedics, The Friendship Hospital of Ili Kazakh Autonomous Prefecture, Yining, 835000, China

**Keywords:** rheumatoid arthritis, TIMP4, interleukin, diagnosis

## Abstract

The current study was designed to explore the clinical significance of serum tissue inhibitor of metalloproteinase 4 (TIMP4) levels in rheumatoid arthritis (RA). The GSE1919 chip was analyzed, differentially expressed genes (DEGs) were identified, and gene ontology as well as Kyoto Encyclopedia of Genes and Genomes analyses of the identified DEGs were conducted. Patients with RA (*n* = 96) and healthy individuals (*n* = 96) were enrolled in this study. Serum from the participants was collected, and RT-qPCR as well as WB have been conducted to examine TIMP4 levels; additionally, interleukin (IL)-6 and IL-1β levels were determined using the ELISA method. Pearson’s correlation analysis was conducted for evaluating relationships between the expression levels of TIMP4 and those of IL-6 or IL-1β. A receiver operating characteristic (ROC) curve was drawn to determine the potential diagnostic value of serum TIMP4 for RA. TIMP4 was identified as a markedly downregulated gene involved in RA development. TIMP4 levels were significantly decreased in patients with RA, and the results of the ROC analysis showed that TIMP4 may be a potential diagnostic marker. Furthermore, the concentrations of IL-6 and IL-1β were markedly elevated in patients with RA. Finally, TIMP4 levels showed negative correlation with the levels of either IL-6 or IL-1β. TIMP4 is downregulated in RA and is a reliable serum marker for RA diagnosis.

## Graphical abstract

**Figure:**
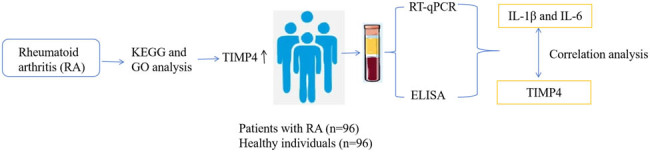

## 1 Introduction

Rheumatoid arthritis (RA) is a systemic autoimmune disorder [1]. It is characterized in that the immune system mistakenly attacks the joint tissue, resulting in persistent inflammatory reaction in the joint cavity [2]. In RA, the abnormal activation of the immune system leads to the aggregation of inflammatory cells such as T cells and macrophages in joints [3,4]. These cells release a variety of inflammatory mediators, such as cytokines and chemokines, which further activate more immune cells and cause angiogenesis and tissue damage [5]. This process leads to inflammation, swelling, and thickening of joint synovium, which produces a lot of synovial fluid, eventually destroying joint structure and causing joint deformation and functional loss [6,7]. In addition, synovitis can also cause systemic immune and inflammatory reactions by releasing inflammatory mediators and chemokines [8]. This systemic reaction may lead to the involvement of other organs and aggravate the overall condition of the patients. T helper cells, a subgroup of T cells, were able to produce cytokines, such as IL-6 and IL-1β, which increases the growth of immune cells and induces chronic inflammation [9,10]. According to clinical epidemiological surveys, the global prevalence of RA is 5–10‰. Patients with advanced RA may suffer from disability, which imposes huge economic burdens on society and severely affects the mental health patients with RA. RA is primarily diagnosed based on clinical manifestations and serological tests [11]. Nevertheless, certain manifestations are often easily confused with other joint diseases. Serological tests mainly refer to autoantibodies, and some laboratory indicators may cause misdiagnosis [12]. Therefore, identifying effective early diagnostic markers for RA remains a long-term task.

Tissue inhibitor of metalloproteinase 4 (TIMP4) belongs to the TIMP family. Previous studies have suggested that TIMPs suppress tumor invasion and metastasis by inhibiting matrix metalloproteinases (MMP) [13]. TIMP4 is considered an anti-inflammatory gene in several diseases [14]. For example, mice lacking TIMP4 showed higher levels of inflammatory cytokines and MMP activity [15]. Additionally, the AMPK/TIMP4 signaling cascade is promoted by zingerone to inhibit inorganic phosphate-induced vascular calcification, thereby inhibiting inflammation in vascular smooth muscle cells [16]. However, whether TIMP4 plays a key role in RA remains unclear. Therefore, we conducted the current study to analyze the serum levels of TIMP4 in patients with RA, and assessed the correlation between TIMP4 and RA inflammatory biomarkers to evaluate the potential of TIMP4 as a novel serological biomarker for RA.

## 2 Materials and methods

### 2.1 Bioinformatics

The gene expression profile data of RA were downloaded from Geodatabase (downloaded from https://www.ncbi.nlm.nih.gov/geo/). The GSE1919 data set, including five Ras and five control groups, was selected, and the differentially expressed genes (DEGs) were screened by using R-packet limma. The adjusted FDR < 0.05 and |fold change| > 1.5 were considered to be statistically significant. The differential expression of mRNA was studied. The adjusted *P* value was analyzed in TCGA to correct the false positive results. Set the threshold as “Adjusted *P* < 0.05 and log2 (multiple change) > 1 or log2 (multiple change) < −1” to screen differentially expressed mRNA.

### 2.2 Gene ontology (GO) and Kyoto Encyclopedia of Genes and Genomes (KEGG) pathway analysis

GO analysis was conducted to predict the biological process (BP), molecular function (MF), and cellular components related to RA DEGs. The KEGG database (https://www.genome.jp/kegg/) was used to analyze the signaling pathways in which the DEGs were enriched in.

### 2.3 Clinical samples

Serum samples (*n* = 96) were collected from patients who were first diagnosed as RA in the Second Affiliated Hospital of Nantong University from January 2022 to December 2023. Control serum samples (*n* = 96) were obtained from healthy individuals who underwent physical examination in the hospital and the demographic criteria matched 96 patients. Patients with cancer, severe metabolic diseases, autoimmune diseases, or cardiovascular diseases were excluded from this study. Serum samples of the participants were promptly stored at −80°C until needed. The diagnostic criteria for RA were based on the American College of Rheumatology/European League Against Rheumatism RA diagnostic criteria, promulgated in 2010.


**Informed consent:** Informed consent has been obtained from all individuals included in this study.
**Ethical approval:** The research related to human use has been complied with all the relevant national regulations, institutional policies and in accordance with the tenets of the Helsinki Declaration, and has been approved by the Ethics Committee of the Second Affiliated Hospital of Nantong University.

### 2.4 Real-time quantitative PCR (RT-qPCR)

TRIzol (Invitrogen) was used to extract RNA from the clinical samples, and then cDNA was synthesized through reverse transcription. RT-qPCR was performed using the Quant One Step qRT-PCR (Probe) Kit (LM-0102; LMAI-Bio Co., Ltd) with the GeneAmp PCR System 9700 (ABI; Thermo Fisher). The expression levels of mRNAs were evaluated by the 2^−ΔΔCt^ method, and GAPDH was used as the reference gene. The primer sequences were as follows: TIMP4 forward 5′-CAGACCCTGCTGACACTGAA-3′ and reverse 5′-AGGGCTCGATGTAGTTGCAC-3′; GAPDH forward 5′-ACCCACTCCTCCACCTTTGAC-3′ and reverse 5′-TGTTGCTGTAGCCAAATTCGTT-3′.

### 2.5 Western blot

RIPA lysis buffer (Beyotime) has been applied for protein extraction, and then the concentration of the proteins was detected by BCA kit, and electrophoresis was performed using 12% SDS-PAGE gel. After electrophoresis (80 V, 2 h), the samples were transferred to PVDF membrane (300 mA, 90 min), blocked by 5% non-fat milk for 1 h, and incubated with primary antibodies overnight. In the following day, the membranes were treated by the horseradish peroxidase-conjugated antibodies, and then the protein bands were detected by ECL reagents with a chemiluminescence detection system. GAPDH was used as the internal control.

### 2.6 ELISA

The levels of IL-6 and IL-1β were measured by ELISA kits (SP10234 and SP10180; both from Wuhan Saipei Biotechnology Co., Ltd). According to the instructions of ELISA kit, the sample is detected, and whether the sample is positive or not is judged according to the criteria of the kit. Calculate the percentage of positive samples, which is the sensitivity. In order to improve the sensitivity of ELISA kit, the following measures are taken: adjusting the reaction temperature, time, and pH value to improve the binding efficiency of antigen and antibody.

### 2.7 Statistical analysis

Statistical analyses were conducted using GraphPad (version 8.2.1.441, GraphPad Software Inc.), and data are presented as mean ± standard deviation. Student’s *t*-test and rank-sum test were used for comparing two groups. Receiver operating characteristic (ROC) curves were constructed to determine the sensitivity and specificity of serum TIMP4 levels for the diagnosis of RA. Pearson correlation analysis and logistic multivariate analysis were used to evaluate the correlation between variables. Differences were considered statistically significant for *p* < 0.05.

## 3 Results

### 3.1 TIMP4 was downregulated in RA

**Figure 1:**
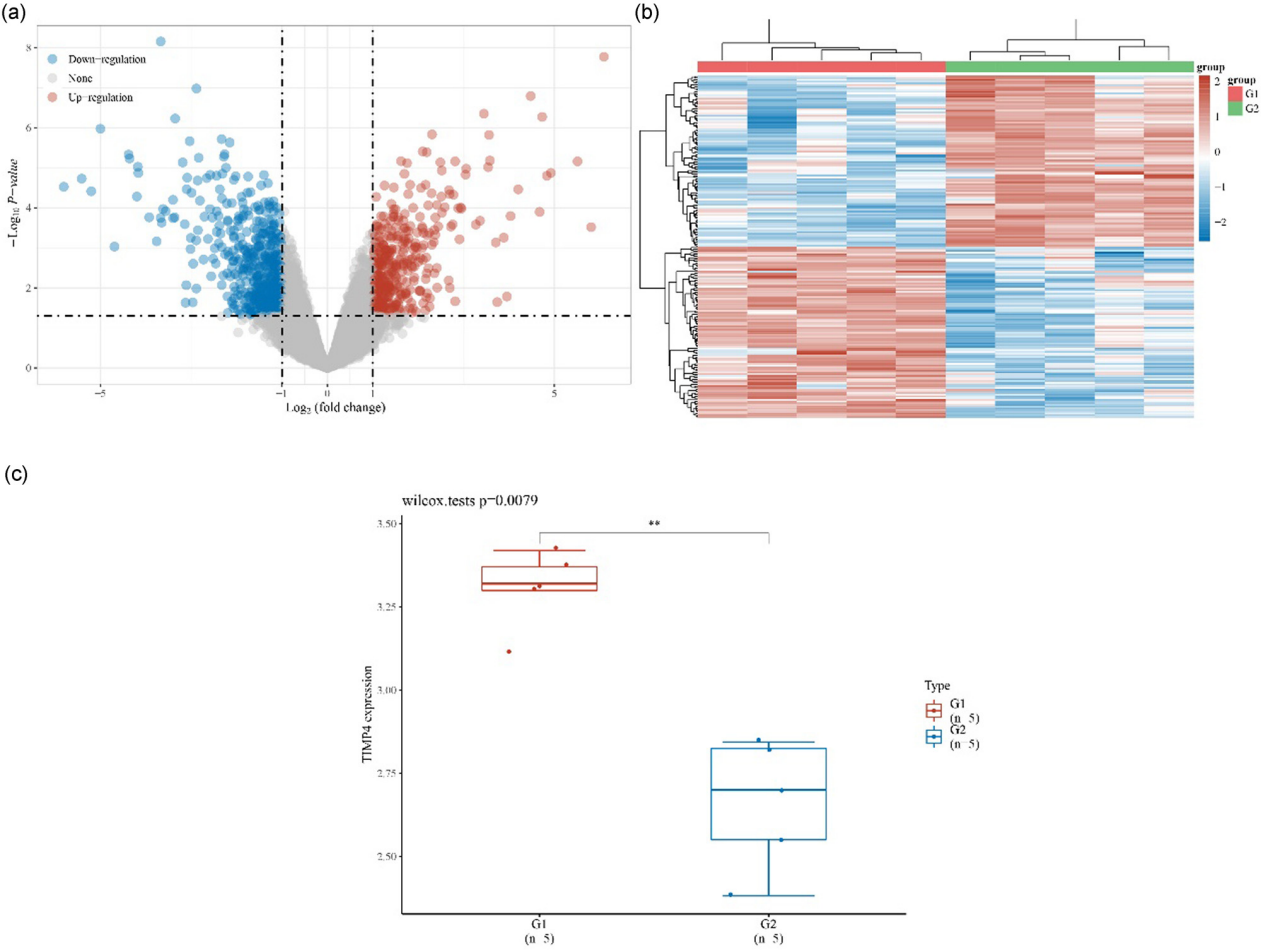
Bioinformatic analysis of RA DEGs. The RA DEGs were distributed in (a) volcano map and (b) heatmap. (c) Expression level of TIMP4 analyzed from gene database GSE1919. **P < 0.01. RA, rheumatoid arthritis; DEG, differentially expressed gene.

DEGs were used to analyze gene microarray profiles between RA and control samples. We found that 364 and 497 DEGs were upregulated and downregulated, respectively (Figure 1a) in the volcano map and heatmap (Figure 1b). The top 20 upregulated and downregulated genes are listed in Table 1. Among the top 20 downregulated genes, TIMP4 was the most markedly downregulated in RA (*p* = 0.0079; Figure 1c).

**Table 1:** Top 20 upregulated and downregulated genes

Gene name	Log FC
**Top 20 upregulated genes**	
JCHAIN	5.80592
CXCL9	5.41172
CXCL10	5.20186
CD27	4.99936
MMP1	4.69088
MMP3	4.37879
LGALS2	4.35772
CXCL6	4.19598
CXCL13	4.17583
GZMK	4.16508
KLRB1	3.92523
GZMB	3.761
GZMA	3.67424
PTPRCAP	3.6701
PCLAF	3.65057
LAMP3	3.61866
CD79A	3.56031
GNLY	3.40081
LRRC15	3.39899
TNFSF11	3.36374
**Top 20 Downregulated genes**	
ZBTB16	−6.093446817
ADIPOQ	−5.811250196
APOD	−5.511514806
FABP4	−4.925550857
PCK1	−4.836592049
ADH1C	−4.732207442
ADH1B	−4.675932971
ANGPTL7	−4.479179556
GPD1	−4.20501228
PLIN1	−4.036929982
DDX3Y	−3.954681864
MYOC	−3.885788571
RPS4Y1	−3.74238006
SLC6A3	−3.711792741
SCD	−3.580080765
FASN	−3.561981707
TIMP4	−3.541400657
MAOA	−3.448798504
LAMA2	−3.363814896

### 3.2 TIMP4 may be functionally associated with RA

**Figure 2:**
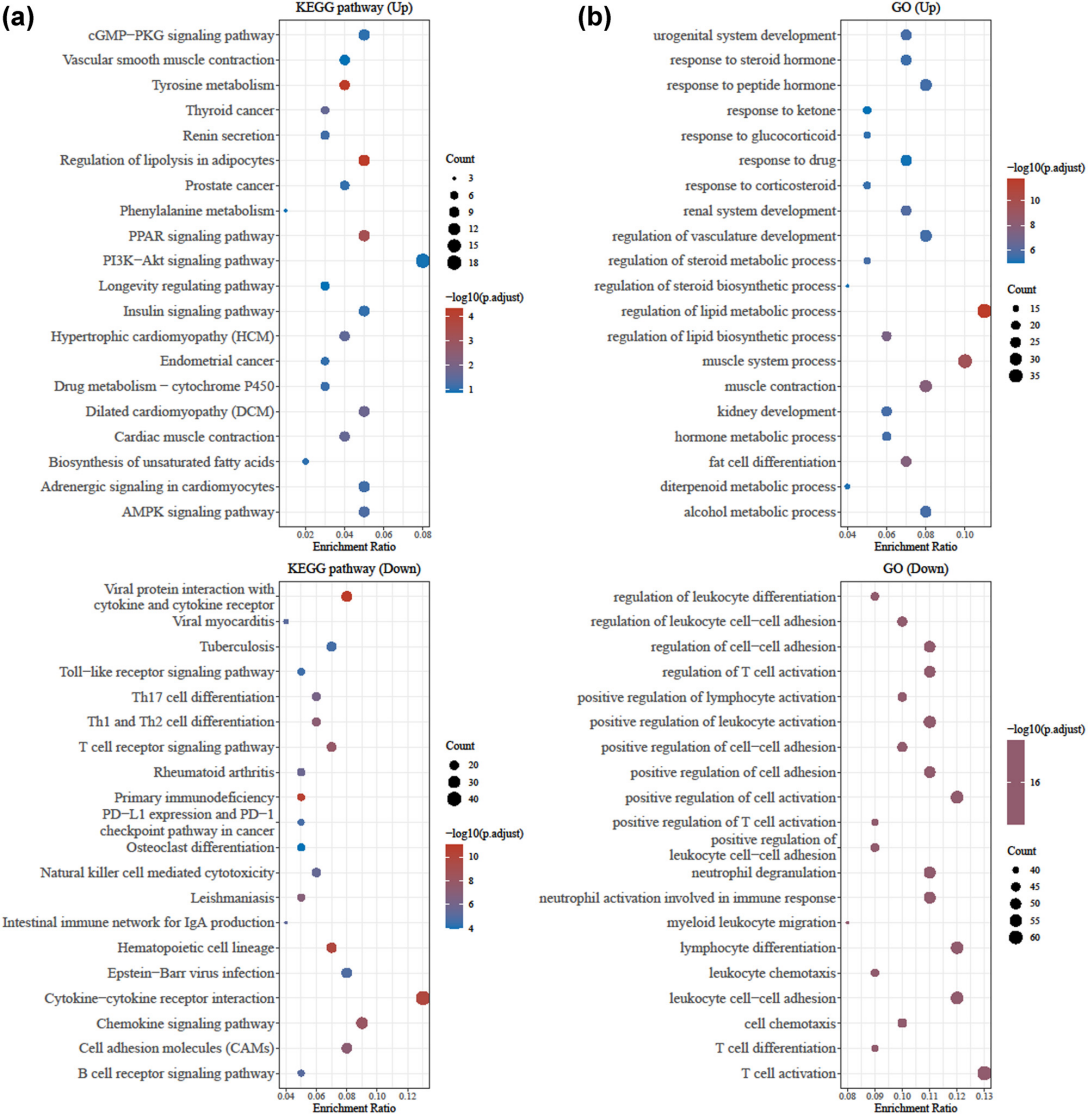
Functional enrichment of RA DEGs. (a) KEGG analysis showed the related signaling pathways enriched by (above) upregulated and (below) downregulated DEGs in RA. (b) GO analysis showed the MFs enriched by (above) upregulated and (below) downregulated DEGs in RA. RA, rheumatoid arthritis; DEG, differentially expressed gene; KEGG, Kyoto Encyclopedia of Genes and Genomes; GO, gene ontology.

Information regarding the RA DEGs was applied to GO term enrichment analysis and KEGG analysis to examine the related MFs and signaling pathways. The PI3K-Akt signaling pathway and regulation of lipid metabolic processes were the most enriched in the upregulated DEGs according to KEGG (Figure 2a) and GO (Figure 2b) analyses, respectively. The KEGG results showed that the downregulated DEGs were abundantly enriched in cytokine–cytokine receptor interaction signaling pathway and RA (Figure 2a). Moreover, the GO analysis showed that the downregulated DEGs were enriched in leukocyte cell–cell adhesion, lymphocyte differentiation, and positive regulation of cell activation, especially T cell activation (Figure 2b). These analyses revealed the important roles of PI3K-Akt signaling pathway and lipid metabolism process in the pathogenesis of RA, and the potential roles of cytokine–cytokine receptor interaction, leukocyte–cell adhesion, lymphocyte differentiation, and T cell activation in RA, suggesting that abnormal expression or dysfunction of TIMP4 may be an important factor in the pathogenesis of RA.

### 3.3 TIMP4 is a potential serum diagnostic marker for RA

**Figure 3:**
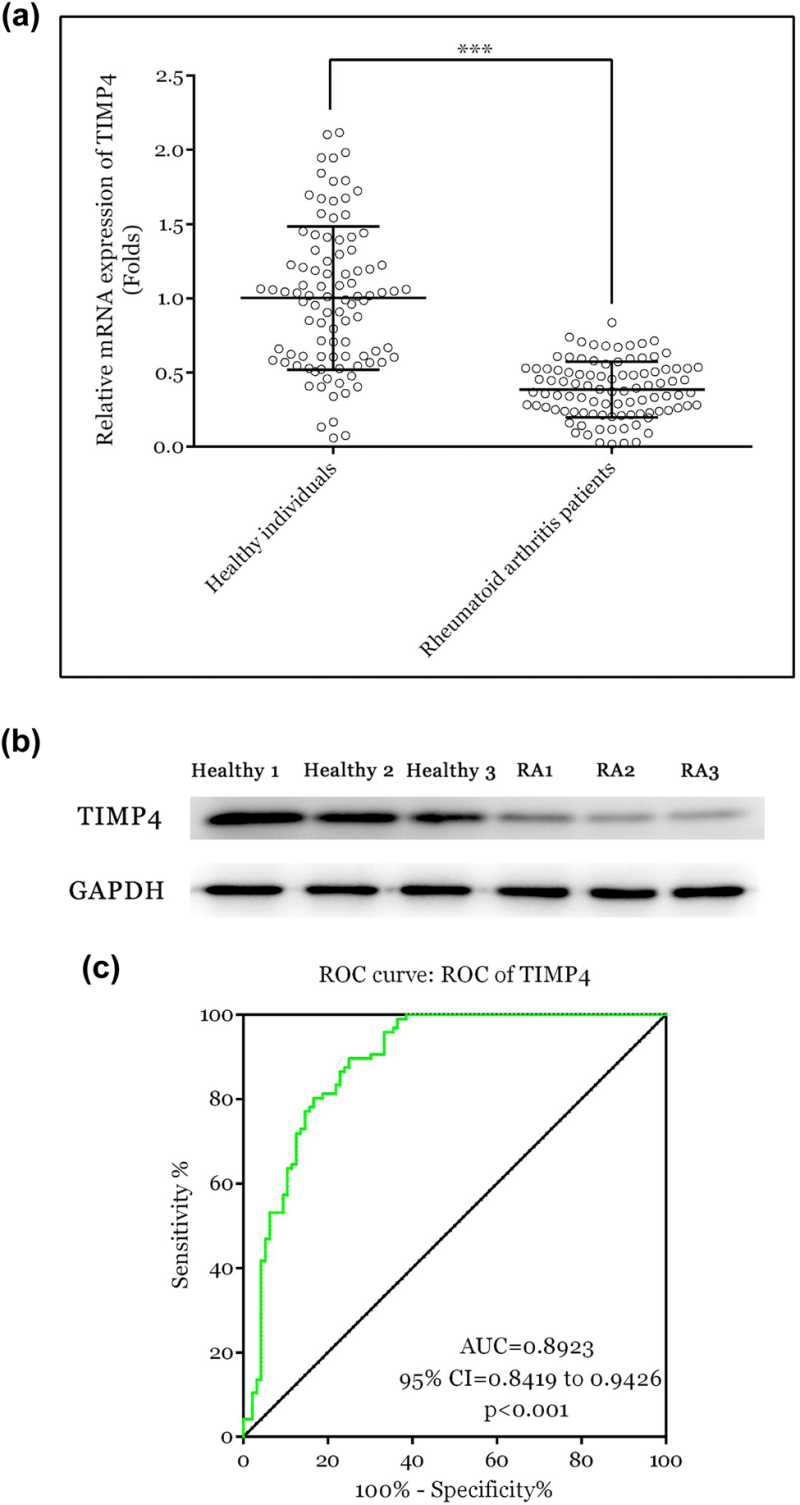
Expression level of TIMP4 was reduced in RA patients. (a) RT-qPCR for TIMP4 in serum of the RA patients (*n* = 96) and healthy individuals (*n* = 96). (b) WB for TIMP4 in serum of the RA patients and healthy individuals. (c) ROC curve of TIMP4 in RA. ***P < 0.001. RA, rheumatoid arthritis; ROC, receiver operating characteristic.

Obtaining synovial tissues from patients and healthy controls for RA diagnosis is difficult in clinical applications. In recent years, the detection of gene expression in circulating RNAs isolated from blood samples has become a new method for disease diagnosis. To further verify the correlation between TIMP4 and RA, TIMP4 expression was examined using RT-qPCR and WB. The clinical information of the patients and healthy controls is shown in Table 1. As Figure 3a and b indicates, TIMP4 expression was lower in patients with RA than in healthy individuals. Subsequently, ROC curve of serum TIMP4 levels was constructed to analyze the sensitivity and specificity for RA diagnosis. The area under the curve (AUC) of TIMP4 was 0.8923 (95% confidence interval: 0.8419–0.9426), implying that TIMP4 is a potent serum marker for RA diagnosis (Figure 3c).

### 3.4 Expression of IL-6 and IL-1β increased in patients with RA

**Figure 4:**
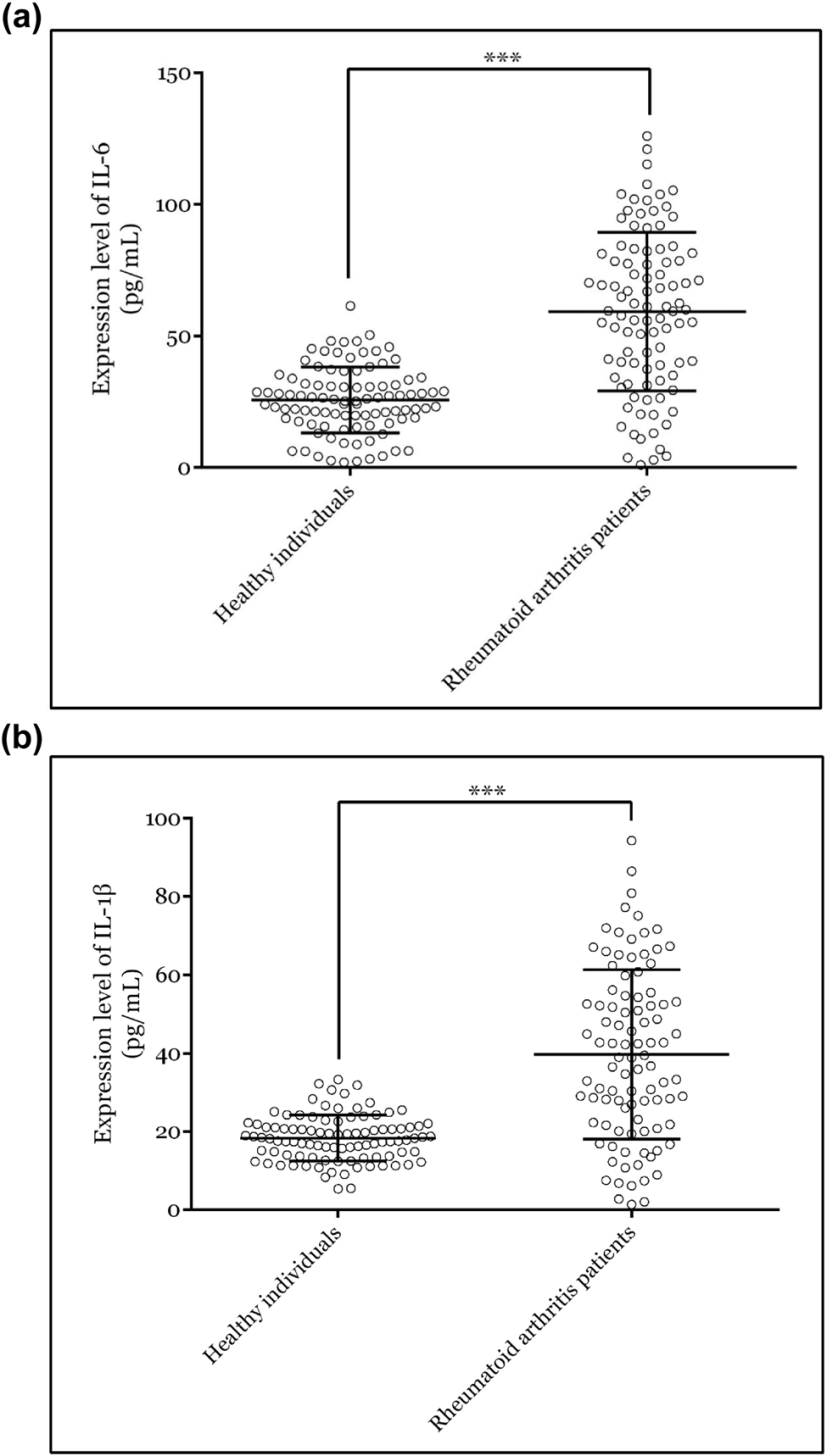
Serum concentrations of IL-6 and IL-1β were promoted in RA patients. The serum concentrations of (a) IL-6 and (b) IL-1β in RA patients and healthy controls were determined by ELISA assay. **P < 0.01; ***P < 0.001. RA, rheumatoid arthritis; IL, interleukin.

RA generally induces the secretion of pro-inflammatory cytokines. Herein, we measured the serum IL-6 and IL-1β levels by ELISA. As expected, both IL-6 and IL-1β levels were markedly increased in the serum of patients with RA than that in the serum of healthy participants (Figure 4a and b).

### 3.5 TIMP4 levels were negatively correlated with either IL-6 or IL-1β in the serum of patients with RA

**Figure 5:**
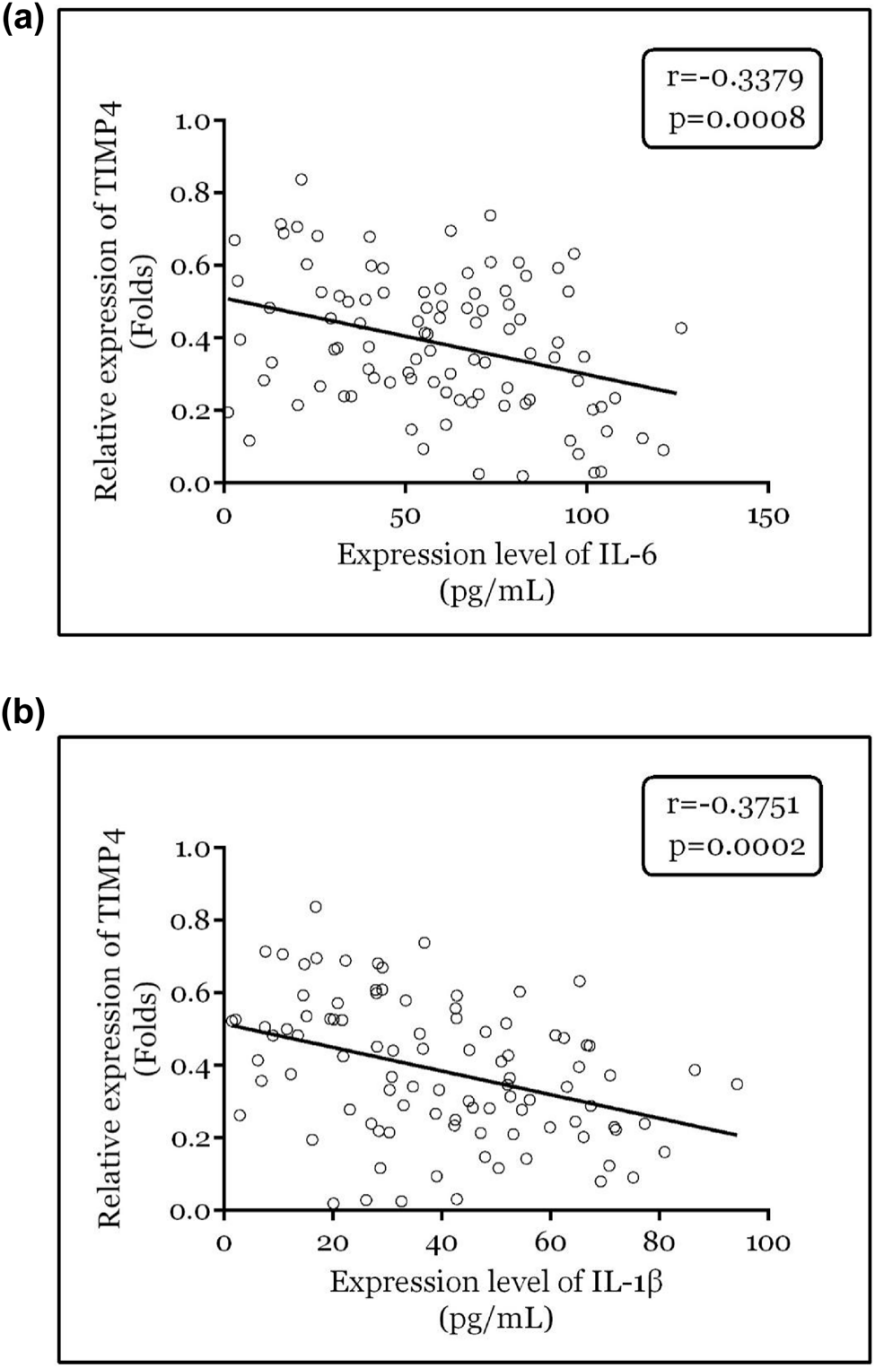
TIMP4 was negatively correlated with IL-6 and IL-1β. Pearson analyses between serum levels of TIMP4 and (a) IL-6, (b) IL-1β.

The correlation between TIMP4 and the levels of IL-6 and IL-1β in serum were calculated. As shown in Figure 5a and b, TIMP4 levels were negatively correlated with the levels of IL-6 (*r* = −0.3379; *p* = 0.0008) and IL-1β (*r* = −0.3751; *p* = 0.0002). At the same time, the relationship between TIMP4 and inflammatory markers was analyzed by multivariate analysis. As shown in Table 2, TIMP4 is closely related to IL-6 and IL-1β.

**Table 2:** Binary logistic regression analysis of the relationship between TIMP4 and inflammatory markers

Variables	SE	95% CI	*P* value
IL-6	0.002332	−0.01188 to 0.002591	0.0027
IL-1β	0.001720	−0.004129 to 0.002726	<0.0001

## 4 Discussion

In the current study, TIMP4 was identified as one of the most significantly downregulated DEG in RA by bioinformatics analysis. Clinically, we observed that TIMP4 levels decreased in the serum of patients with RA. A negative correlation was observed between TIMP4 and inflammatory cytokine levels. Therefore, these results indicate the diagnostic potential of TIMP for RA.

TIMP4 is a member of the TIMPs protein family and is involved in BP such as cell growth and metastasis [15]. Previously, TIMP4 has been demonstrated to be upregulated in breast cancer [17], prostate cancer [18], glioblastoma [19], and head and neck squamous cell carcinoma [20]; whereas, low TIMP4 levels have been observed in patients with pancreatic cancer. However, the role of TIMP4 in joint diseases remains poorly understood. IL-17A-negative patients with RA showed lower MMP1/TIMP4 and MMP3/TIMP4 ratios in post anti-TNF-α therapy, which provided an initial clue for the diagnostic potential of TIMP4 for RA [21]. TIMP4 was markedly decreased in cartilage samples of femoral heads obtained from patients with osteoarthritis (OA) [22]. A similar decrease has been observed in patients with primary hip OA. Diani et al. showed that TIMP4 levels were notably elevated in patients with psoriatic arthritis receiving systemic treatment compared to patients with psoriasis, with good diagnostic accuracy (AUC > 0.7) [23]. In the present study, we identified TIMP4 as the most markedly downregulated gene in RA using bioinformatic analysis. Moreover, functional predictions showed that both T cell activation and the RA signaling pathway were enriched in the DEGs that were downregulated in RA. PI3K-Akt signaling pathway and regulation of lipid metabolism in RA are significantly upregulated, which may promote synovial cell proliferation and inflammation. However, cytokine–cytokine receptor interaction, graphene oxide, and leukocyte–cell adhesion are downregulated, reflecting the imbalance of immune regulation. These findings suggest that abnormal expression or dysfunction of TIMP4 may be an important factor in the pathogenesis of RA. By analyzing TIMP4 levels in the serum of patients with RA and healthy individuals, we found that TIMP4 was notably inhibited in patients with RA, which is similar to the results of previous studies. Furthermore, the ROC results suggested that TIMP4 is a reliable diagnostic marker for RA owing to its high sensitivity and specificity. Taken together, we demonstrated that TIMP4 is downregulated in RA and could serve as an RA predictor.

Nonspecific inflammation initiates RA, followed by T-cell activation and chronic inflammation. During chronic inflammation, cytokines that exert pro-inflammatory functions tend to be released excessively [24]. IL-6 increases the levels of VEGF under the action of the soluble IL-6 receptor, thereby changing the permeability of blood vessels and leading to the formation of RA pannus [25]. IL-1β mainly inhibits the repair of bone and cartilage, which leads to bone destruction in patients with RA [26]. A clinical study indicated that the degree of osteoporosis in patients with RA was positively correlated with IL-6 levels [27]. Moreover, a cross-sectional study revealed that severe disease activity was correlated with serum IL-6 levels in patients with RA [28]. In the present study, we found that the serum levels of IL-6 and IL-1β in patients with RA were considerably increased, which is consistent with previous studies. Furthermore, serum TIMP4 levels were negatively correlated with the serum levels of both IL-6 and IL-1β, further demonstrating the diagnostic roles of TIMP4 in RA.

## 5 Conclusion

Taken together, our findings are the first to propose TIMP4 as an efficient serum marker for RA diagnosis and predictor of RA-induced inflammation. However, the present study has some limitations. First, the clinical sample included only a few participants (*n* = 96), and more extensive research is needed in the future. Finally, we only performed serological analysis and focused on the clinical significance of TIMP4 in RA. The underlying mechanism should also be investigated by performing cell and animal studies.
